# Body –to-head transplant; a "caputal" crime? Examining the corpus of ethical and legal issues

**DOI:** 10.1186/s13010-018-0063-2

**Published:** 2018-07-13

**Authors:** Zaev D. Suskin, James J. Giordano

**Affiliations:** 1 Harvard Medical School Center for Bioethics, 641 Huntington Avenue, Boston, MA 02115 USA; 2 Georgetown University School of Medicine, 3900 Reservoir Road NW, Washington, DC, 20057 USA; 3 Neuroethics Studies Program, Pellegrino Center for Clinical Bioethics, Bldg. D, Rm 238, 4000 Reservoir Road NW, Washington, DC, 20057 USA; 4 Departments of Neurology and Biochemistry, Georgetown University Medical Center, 3900 Reservoir Road NW, Washington, DC, 20057 USA

**Keywords:** Body-to-head transplant (BHT), HEAVEN, Transplant, Neuroethics, Bioethics, Global ethics, Neurosurgery

## Abstract

Neurosurgeon Sergio Canavero proposed the HEAVEN procedure – i.e. *he*ad *a*nastomosis *ven*ture – several years ago, and has recently received approval from the relevant regulatory bodies to perform this body-head transplant (BHT) in China. The BHT procedure involves attaching the donor body (D) to the head of the recipient (R), and discarding the body of R and head of D. Canavero’s proposed procedure will be incredibly difficult from a medical standpoint. Aside from medical doubt, the BHT has been met with great resistance from many, if not most bio- and neuroethicists.

Given both the known challenges and unknown outcomes of HEAVEN, several important neuroethical and legal questions have emerged should Canavero be successful, including: (1) What are the implications for transplantology in the U.S., inclusive of issues of expense, distributive justice, organizational procedures, and the cost(s) of novel insight(s)? (2) How do bioethical and neuroethical principles, and legal regulations of human subject research apply? (3) What are the legal consequences for Canavero (or any other surgeon) performing a BHT? (4) What are the tentative implications for the metaphysical and legal identity of R should they survive post-BHT? These questions are analyzed, issues are identified, and several solutions are proposed in an attempt to re-configure HEAVEN into a safe, clinically effective, and thus (more) realistically viable procedure.

Notably, the permissibility of conducting the BHT in China fosters additional, important questions, focal to (1) whether Western ethics and professional norms be used to guide the BHT – or any neuroscientific research and its use - in non-Western countries, such as China; (2) if the models of responsible conduct of research are identical, similar, or applicable to the intent and conduct of research in China; and (3) what economic and political implications (for China and other countries) are fostered if/when such *avant garde* techniques are successful.

These questions are discussed as a further impetus to develop a globally applicable neuroethical framework that would enable both local articulation and cosmopolitan inquiry and oversight of those methods and approaches deemed problematic, if and when rendered in more international settings.

## Main text

“Success shall crown my endeavours. Wherefore not? Thus far I have gone, tracing a secure way over the pathless seas... Why not ... proceed over the untamed yet obedient element? What can stop the determined heart and resolved will of man?”

- *Mary Shelley* [1]

When Mary Wollencroft Shelly wrote these words in 1818, she had no idea that exactly 200 years in the future, her fictional Dr. Frankenstein’s endeavors would be so akin to what neurosurgeon Sergio Canavero views as the the crowning culmination of his life’s work. Canavero has recently made headlines by planning to perform the first human body-to-head transplant (BHT) in China during the coming year. By definition, the BHT procedure involves attaching the donor body (D) to the head of the recipient (R), and discarding the body of R and head of D. Canavero, who has now performed the procedure on two cadavers [2], likens himself to famous, fearless, and forward-thinking aeronautical pioneers, analogously and proverbially keeping his eyes on the stars. In this light, Canavero calls the procedure HEAVEN [3], i.e. the *he*ad *a*nastomosis *ven*ture; and the operation is being viewed as exciting and inspiring, as well as with doubt, scorn and resistance.

Bioethicist Arthur Caplan, has called HEAVEN “rotten scientifically” and “fake news” that merits “contempt and condemnation.” [4] A recent issue of the *American Journal of Bioethics - Neuroscience* [5] was devoted in its entirety to addressing the procedure, and was rife with both scientific and ethical criticism. It is not surprising that like many (if not most) innovations that are ahead of their time, HEAVEN is being met with abundant skepticism. Historically, such negative criticism has been particularly vehement toward other new and often untried methods and procedures of transplantation. For example, when Richard Lawler performed the first kidney transplant in 1950, he was professionally shunned before ultimately achieving clinical success where others had failed [6]. Similarly, Christiaan Barnard, the surgeon who completed the first heart transplant, was told that what he was attempting was unnatural and impossible. His endeavors were also rewarded by eventual success.

The idea of a “head transplant” has been popularized as the stuff of fiction. In Greek mythology, the Minotaur (technically an accursed chimera) was composed of the body of a man and the head of a bull. In the film *Mars Attacks!* [7], head transplants were performed between aliens and humans. The much discussed film *Get Out* [8] centers around a neurosurgeon who performs “brain transplants.” While fictional accounts may be entertaining, BHTs have also been attempted in animal models, often with provocative results. In 1908, Alexis Carrel and Charles Claude Guthrie were able to preserve reflexes in a canine BHT; and during the 1970s, Robert White (who Canavero has claimed was an inspiration for his own work) performed BHT procedures on primates with some success. As recently as 2012, Xiaoping Ren (Canavero’s latest collaborator in the planned attempt to realize the HEAVEN procedure at Harbin Medical University in China) was able to maintain blood supply to the brain in a BHT in mice that survived for 6 months post-operatively [9].

Canavero’s proposed procedure will be incredibly difficult: demanding that the recipient’s cerebral bloodflow be maintained in order to avoid imminent brain damage from hypoxia; requiring meticulous re-attachment of the spinal cord to preserve neurologic function important for both keeping autonomic functions of the body intact and for providing input to the brain, which many have argued is vital for what is referred to as “embodied consciousness;” and necessitating extensive and life-long immunosuppression to prevent transplant rejection. While doubt and considerable caution may be warranted, why the resistance? Why is the term ‘monster’, typically applied to Frankenstein’s creation, now being associated with the creator? The abundant ethical and legal questions are likely to provide an answer.

In the United States, transplant practices are governed by the United Network for Organ Sharing (UNOS) [10], an organization established by Congress in 1984 to effectively address the need for organs by maintaining donor databases, establishing waitlist and matching criteria, and monitoring methods used. In the past year, 2853 transplants were performed, but over 115,000 people still await donor organs [11]. It has been estimated that a single donor could provide organs capable of treating eight recipients [11]. Given this ratio of transplantable organs to patients affected, we could ask why R should receive the entire body of D if D’s organs can be justly distributed to save seven more lives? Current waitlist criteria do not specify the number of organs a recipient patient needs, and patients are put on each organ waitlist separately. But the viability and criteria for the use of several organ systems, such as that of a “whole body” transplant are not currently specified; are new waitlist criteria and definitions needed?

The costs of transplants can be exorbitant. For example, the average cost of a kidney transplant (i.e. the most common organ transplant) is $400,000 [12], whereas a single BHT would involve approximately 80 surgeons and has been estimated to incur costs of $10–100 million [6]. Might not these resources be better spent on funding more transplants and/or developing synthetic organs to meet shortages? On the other hand, Canavero’s procedure, even if not completely successful, could surely yield important information about neurological transplantation, the brain-body relationship, and perhaps even those ways that a brain might be maintained absent a body. Is such information worth the investment? And what if BHTs were privately funded? The National Organ Transplant Act of 1984 prohibits the sale and purchase of organs [13], but there is new debate about the constraints that such laws may incur in light of increasing shortages of viable organs. Will BHTs add to, or mitigate such shortages? And, given the excessive cost of a BHT, will HEAVEN be just for the rich? Indeed, the costs of developing HEAVEN will be enormous and will likely require individual and institutional support. Should UNOS therefore examine the need to develop policies that will consider ‘body waitlists’ so as to ensure that availability/matching is not merely dependent on socioeconomic status?

If Canavero is to “pioneer a new way, explore unknown powers, and unfold to the world the deepest mysteries of creation,” [1] as he has claimed, UNOS will not provide the only oversight for his investigative operation. While no state or federal agency regulates new surgical procedures [14] (unlike the Federal Drug Administration’s authority to regulate novel drugs and medical devices), various laws, treatises, and institutional review boards oversee research conducted with human subjects. Concerns about the probity of research practices became paramount following the atrocities committed by scientists and physicians in Hitler’s Germany, and ultimately resulted in The Nuremberg Code [15] and the Universal Declaration of Human Rights [16]. The doctrines of the *Belmont Report* [17] regarding the ethical treatment of human subjects in biomedical research were codified into law by 45 CFR part 46 in 1978 [18]. The basic tenets require that research should be medically appropriate, have a reasonable chance of success, minimizes risks, and that adequate informed consent be obtained. Prior to engaging human trials, animal studies are typically undertaken; but such evidence regarding the benefits, burdens, and alternatives of a human BHT is still overwhelmingly lacking, making it difficult to follow on the heels of Carrell and Guthrie’s, and White’s research with animals’ heads.

This is why Canavero has relied heavily on the precepts of informed consent [19]. Similar to the constructs advanced in support of recent “right-to-try” legislation, Canavero believes that patients suffering life-threatening bodily illnesses should be able to undergo the experimental surgery with minimal (albeit complete) information, including being informed about the unknowns. Should there be “socially imposed normative limits to rational consent?” [20]. Is “one man’s life or death… but a small price to pay for the acquirement of the knowledge [which we seek?]” [1]. Many argue that a caveat emptor approach to informed consent is insufficient because the risks (e.g. of death or durable suffering beyond that of the pre-operative state) are too great, and the realization of the intended benefits (of the procedure actually working) are highly improbable, if not impossible. Therefore, perhaps a more pertinent question is whether a patient can consent to be killed. Common law maintains that consent is not typically a defense for homicide. But we believe R can authorize his own death for three reasons. First, R is not intending to die, but rather to be temporarily placed in a state in which there is cessation of bodily function and requiring total life support (similar to Barnard’s use of potassium in heart transplants). Second, exceptions exist; for example, voluntary euthanasia is currently illegal, but has moral standing and can incorporate medical procedures. Third, inducing the cessation of bodily functions is procedurally required to attain the intended benefit of the BHT. But Canavero has made outlandish claims regarding the anticipated benefit of the BHT, including predicting a “90+ percent chance of success” [6] and promising that the patient will ability to walk and be able to engage intimate relations again.

Clearly, the BHT will not be permitted to be undertaken in the United States. But what if Canavero *were* to perform such a procedure in the U.S.? Would he face criminal charges? The HEAVEN protocol necessitates that R be “killed” (albeit and hopefully temporarily), as the Uniform Declaration of Death Act [21] defines death as the “irreversible cessation of circulatory and respiratory functions; or irreversible cessation of all functions of the entire brain, including the brainstem.” At the time of decapitation, perfusion to R’s head and D’s body would be maintained, but their hearts and brains would cease to function, respectively. As such, legal scholar Nita Farahany has stated that “it seems as if active euthanasia could be the most lenient characterization of a surgery involving decapitation… [or] could be viewed as intentional or reckless homicide…” [22]. However (and as partially acknowledged by Farahany), this characterization may be erroneous for several reasons. First, D is not being “killed” at all, having been declared (at least) brain-dead pre-operatively. Second, the organ/system cessation of R is purportedly temporary (again, as is common in other types of medical procedures), with death being an unintended and adverse outcome (again, commonly accepted in other medical procedures).

Contrarily, some may attempt to defend and justify Canavero’s actions via the ‘Principle of Double Effect’ – a moral doctrine permitting an otherwise untenable action (and outcome) when achieved via a legitimate act. This doctrine has several key criteria: the action itself must be morally good or neutral; the bad effect must not be the means by which the good effect is achieved; the actor cannot intend the bad effect; and the bad effect must be proportionate to the good effect. This principle is typically instantiated in debates regarding the permissibility of abortion via hysterectomy, or in cases of terminal palliative sedation. However, it seems clear that the BHT would not satisfy all of these criteria: with the “bad effect” (i.e. temporary cessation of bodily function) being the intended means by which the “good effect” (i.e. completed transplant and restoration of bodily function) is achieved.

In any case, it is unclear what repercussions Canavero would face. Far more interesting are the consequences for the patient:“Who was I? What was I? Whence did I come? What was my destination?” [1] One can easily imagine R waking up, looking down at an unfamiliar body, and asking the same questions as Dr. Frankenstein’s fictional creation. The most intriguing question regarding the BHT has been the identity of the person waking up – will they be R or have some embodied sense of being D? Or, perhaps, will they have a subjective experience of being something different? There have been longstanding discussions and debates about the nature of identity. For many, the question of “who am I?” is evolving and evasive. Neuroethicists and philosophers addressing the implications of the BHT have attempted to answer “who” R will be based on modern philosophical and neuro-cognitive theories of the “self.” [23–25] But until (or unless) R awakens and can relate the post-operative phenomenological experience of having a different body, this remains only speculation.

In order to query what a BHT “feels like,” the patient would need to not only live, but also retain consciousness, communicative ability, and memory of their prior embodied experience. While Canavero may not be worried about people remembering him, this is untrodden territory, and if the patient can’t remember who he or she is, we may never know what the pre- versus post-surgical experience is like in subjective terms. And R’s significant memory loss must be carefully considered given the primary procedural risk of HEAVEN is brain hypoxia, with the hippocampus – the part of the brain responsible in large part for memory functions –most prone to anoxic injury. In which case, how do we identify our amnestic anybody?

While the law does not establish a concrete definition of identity, two methods are currently used – one physical and one functional. Physically, DNA is commonly used in a host of identification practices, including in criminal evidence and in paternity testing. But DNA is non-definitive, as *identical* twins share 99.99% similarity; this has already been problematic when identifying the culprit in the cases of a jewelry theft [26] and rape of a nine-year-old girl [27]. Moreover, problems arise as R’s head will have different DNA than his new body.

Alix Rogers elegantly argues that the law has typically taken a functionally “neurocentric” view of identity [28]. Rogers uses the example of conjoined twins – two heads *(*viz. *“caputs”)* sharing the same body (viz. *corpus)* – to show that in such a case, the government still recognizes the existence of two people with distinct identities and rights to self-determination. Furthermore, commonly held views of personhood that rely on the ability to feel pain, including those used in debates about abortion, are also neuro-centric. And if neither of these views seem sufficient, perhaps R should simply be treated (legally) as the same person as before, consistent with other conceptualizations of identity in persons with memory disorders. The law already treats amnestic patients – those who do not retain past memories, cannot form new ones, and/or behave entirely differently following, for example, traumatic brain injury or contraction of a memory disorder – as the same person prior to amnesia (even if socially they may be treated distinctly).

Legal identification is vital because the implications, including citizenship, inheritances, and assets, extend to others – e.g. marriage, parenthood, debts, and wills. Therefore, two things need to occur. First, the legal system must establish a clear definition of identity. Second, until this is done, identity must be established prior to the BHT. At first glance, this would require: (1) that R must agree to preserve their prior legal identity (to account for old responsibilities and adopt new DNA); (2) that R cannot be held in any way accountable for civil, criminal, and contractual responsibilities of D (e.g. paternity); and (3) that D’s healthcare proxy and family must forgo all claims to D’s body.

Even if legal identity can be established, how will R incorporate a new body into the ‘old self?’ The patient may struggle, going through life “just not feeling like their self.” Similar concerns originally plagued surgeons performing face and hand transplants. But evidence has shown that these transplant recipients actually feel more like themselves (i.e. renewing their pre-illness identity) and/or obtain a more complete sense of agency (i.e. regaining lost capacity) after the operation because they can engage public life without the stigma of their prior appearance (e.g. in the case of face transplantees), as well perform previously lost physical functions (e.g. in the case of limb transplantees) [29]. But receiving an entirely new body may be a very different experience. Canavero is not insensitive to these possibilities and issues, and has suggested that advancing certain technologies, such as the use of virtual reality, may allow R to incrementally adapt to the novelty of a forthcoming self in preparation for the BHT. To be sure, extensive pre- and post-transplant psychological counseling must also be provided; a contingency that Canavero has, in fact, recognized and called for.

However, it must be noted that these assertions are rendered in the context of the U.S. legal system and reflect a Western perspective. Canavero intends – and has been authorized – to undertake the BHT in China – where culture, ethics, and law(s) differ not only from the U.S., but from neighboring Asian countries as well. The expanding neuroscientific enterprise in China – perhaps the fastest growing share of the neuroscience market, predicted to reach $34.8 billion by 2024 [30] – as well as guidelines and policies that direct and govern research and medicine in China are in some ways distinct, and more lenient than those in the U.S., Europe and many other countries. Under such open regulatory statues, neuroscientific research, technological development, and their application(s) in biomedicine may advance more freely and rapidly in an explicated “spirit of discovery.” Should Western ethics and professional norms be used to guide the BHT – or any neuroscientific research and use - in China? Imposing Western moral and professional ideologies on China may undermine the history, principles, values, and needs of the Chinese people, as well as hinder the scientific, technological, and economic development of Chinese society [31].

But simple moral (and medical) relativism may also be untenable. The *American Journal of Bioethics-Neuroscience* peer commentaries, and numerous BHT-‘themed’ articles and op-eds in the popular media have focused on the history of human rights’ violations in China and the lack of sufficient research delineating patient risks;. But in the main, such writings have generally failed to ask whether the models of responsible conduct of research, informed consent, and the neuroethical principles underlying their analyses are identical, similar, or applicable to the intent and conduct of research in China.

Consequences of performing the BHT in China extend beyond those of individual patient harm [31], and incur issues, questions, and problems of research- and medical-tourism. It will be important to consider the effect of a ‘brain-drain’ of scientists and physicians from more conservative countries who seek to opportunize professional ethics and rules that are more permissive than those of their home countries. And what if Canavero’s endeavors are successful? Will will his scientific findings and neurosurgical capabilities instigate the viability of BHTs on a broadening scale? Answers to such questions have been made opaque by the scientific, medical, and ethical communities insufficiently appreciating the global inter-connectedness of (and repercussions for) their fields and humanity writ-large, focusing instead and somewhat more parochially on attempts to apply nation-specific and culturally narrow frameworks to globally relevant and influential issues. Such a stance may have far graver consequences than “missing the mark” (partially or entirely) – brain sciences and discoveries could be stagnated, beneficial patient outcomes proscribed (and adverse effects permitted), and a large number of inextricably connected communities could be ill-prepared to work together and interpret and manage the consequences of their and others’ work.

Perhaps, more of a ‘middle-ground’ or, rather a more globally applicable stance must be found for the professional ethics informing international laws relevant to Canavero’s procedure (and other cutting edged, if not *avant garde* use of methods and tools). Previously, we have proposed a risk assessment and mitigation approach, and set of principles that could be utilized to leverage neuroethical analyses and guidance, which could be applicable to both local and global contexts [32–35]. By asserting “standards of objectivity sufficient for broadly justifying practical ethical positions” on the twenty-first century world stage, such frameworks would allow Chinese culture, research, medicine, and patients to flourish, while permitting overseas oversight and inquiry into those methods and approaches that are problematic, if and when rendered in more international settings.

## Conclusion

The capability and potential of current and emerging neuroscientific tools and techniques may well harken Shelley’s query, “With how many things are we on the brink of becoming acquainted, if cowardice or carelessness did not restrain our inquiries?” [1]. Canavero’s endeavor to perform the first BHT is exciting, provocative, problematic, and evidently contentious. Given the current incentives for advancing the capabilities of neuroscience and technology in medicine, his claims (and stated commitment) to lessen the burden of debilitating neurological disease should be seen as a “shot across the bow,” a portend of things to come. Thus, we believe that his claims, and the palette of emerging neuroscientific capabilities should be met with thorough consideration, appropriate concern and acknowledgment of trajectories for (both positively and negatively valent) capitalization, and not simply condemnation or *laissez faire* concession. Indeed, it is equally important to heed – and avoid – carelessness. If Canavero and his enthusiasts do not want the development of a BHT to be de-capitated, then deep and appropriately prudent deliberations and measures must be taken now, and used to develop consistent metrics for if and when such a procedure can safely, and should (with probability of some genuine success) be attempted. Such an agenda should insure that further animal studies are conducted and subject to peer review. And observation and scrutiny of any such work, and regard for its realistic translation to a human trial, should be encouraged, supported, and welcomed.

Canavero has been called a “cowboy”; and if that is a fitting title, it should demand a proverbial “white hat” atop a white lab coat. If the intent is to benefit patients, the process of informed consent must be undertaken with greater humility and utmost stringency. UNOS – which has claimed to recognize at least the meritable intent of a BHT – should be engaged to establish guidelines that direct and govern the type and extent of preliminary research necessary to provide sufficient (or at least satisficing) “medically-based evidence” to translate the procedure to human application. All in all, numerous medical, ethical, and legal steps must be taken globally before HEAVEN can be realized on earth. If and when such steps are taken, success will not just crown Canavero’s endeavors, but will propel – and sustain – the right and good use of neuroscience in what may be an inevitable, and we hope inspiring, march forward.

## Abbreviations

BHT: body-to-head transplant
D: donor
DNA: deoxyribonucleic acid
R: recipient
UNOS: United Network for Organ Sharing
